# The Process of Transformation–The Core of Analytical Psychology and How it Can Be Investigated^1^

**DOI:** 10.1111/1468-5922.13095

**Published:** 2025-05-09

**Authors:** Christian Roesler

**Affiliations:** ^1^ Freiburg Germany

**Keywords:** archetype theory, process research, psychotherapy process, Structural Dream Analysis, transformation in psychotherapy, théorie des archétypes, processus de psychothérapie, recherche sur les processus, transformation en psychothérapie, Analyse Structurelle des Rêves, Archetypentheorie, Psychotherapieprozeß, Prozeßforschung, Transformation in der Psychotherapie, Strukturelle Traumanalyse, teoria degli archetipi, processo psicoterapeutico, ricerca sul processo, trasformazioni in psicoterapia, Structural Dream Analysis, теория архетипов, психотерапевтический процесс, исследование процесса, трансформация в психотерапии, структурный анализ сновидений, teoría del arquetipo, proceso psicoterapéutico, investigación de procesos, transformación en psicoterapia, Análisis Estructural de Sueños, 原型理论, 心理治疗过程, 过程研究, 心理治疗中的转化, 结构性梦境分析

## Abstract

This article presents the scientific background for the introduction of the international conference in Freiburg (“The Process of Transformation”, Catholic University Freiburg, October 4–6, 2024). On the basis of a thorough analysis of archetype theory, it is first established that there is confusion regarding the definition of archetypes in analytical psychology. In fact, Jung’s archetype theory contains not one, but four distinct theoretical strands. While Jung’s biologistic and anthropological arguments in particular must be regarded as refuted, the idea of a universal form of a transformation process in psychotherapy is examined in more detail. It was Jung’s endeavour to create a map of this universal transformation process in order to inform psychotherapeutic work. However, there is a glaring lack of solid research on Jung’s conceptions and a concerted effort is called for in analytical psychology to develop appropriate research strategies. As an example of such research, results from the Structural Dream Analysis research project are reported.

## Abstract (fr)

Cet article présente le contexte scientifique de l'introduction de la conférence internationale sur le « Processus de transformation » déroulée à Fribourg, Allemagne, en octobre 2024. Sur la base d'une analyse approfondie de la théorie des archétypes, il est tout d'abord établi qu'il existe une confusion quant à la définition des archétypes dans la psychologie analytique. En fait, la théorie des archétypes de Jung ne contient pas un seul, mais quatre courants théoriques distincts. Alors que les arguments biologistes et anthropologiques de Jung doivent être considérés comme réfutés, l'idée d'une forme universelle de processus de transformation en psychothérapie est examinée plus en détail. Jung s'est efforcé de créer une cartographie de ce processus de transformation universel afin d'informer le travail psychothérapeutique. Cependant, il y a un manque flagrant de recherches solides sur ces conceptions de Jung, et un effort concerté est nécessaire en psychologie analytique pour développer des stratégies de recherche appropriées. Les résultats du projet de recherche sur l'analyse structurelle des rêves sont présentés à titre d'exemple d'une telle recherche.

## Abstract (de)

Dieser Artikel stellt den wissenschaftlichen Hintergrund zur Einführung der internationalen Konferenz zum "Prozeß der Transformation" vor, die im Oktober 2024 in Freiburg stattfand. Auf der Grundlage einer gründlichen Analyse der Archetypentheorie wird zunächst festgestellt, daß hinsichtlich der Definition von Archetypen in der Analytischen Psychologie Verwirrung herrscht. Tatsächlich enthält Jungs Archetypentheorie nicht einen, sondern vier verschiedene theoretische Stränge. Während insbesondere Jungs biologistische und anthropologische Argumente als widerlegt gelten müssen, wird die Idee einer universellen Form eines Transformationsprozesses in der Psychotherapie genauer untersucht. Jungs Bestreben war es, eine Karte dieses universellen Transformationsprozesses zu erstellen, um die psychotherapeutische Arbeit zu unterstützen. Es besteht jedoch ein eklatanter Mangel an solider Forschung zu diesen Konzeptionen Jungs und es bedarf einer konzertierten Anstrengung in der Analytischen Psychologie, um geeignete Forschungsstrategien zu entwickeln. Als Beispiel für eine solche Forschung werden Ergebnisse des Forschungsprojekts Strukturelle Traumanalyse vorgestellt.

## Abstract (it)

Questo articolo passa in rassegna il panorama scientifico che ha fatto da introduzione alla Conferenza internazionale su "Il processo di trasformazione", che ha avuto luogo a Freiburg, nel mese di ottobre 2024. A partire da una attenta analisi della teoria deli archetipi, è stato chiarito che vi è una certa confusione a riguardo della definizione degli archetipi, nella psicologia analitica. Infatti, la teoria junghiana deli archetipi contiene non solo uno, ma ben quattro distinti filoni teorici. Mentre le argomentazioni junghiane di tipo biologico ed antropologico sono state confutate, l'idea di una forma universale del processo di trasformazione in psicoterapia è considerata con più attenzione. L'obiettivo di Jung è stato quello di definire una sorta di mappa di questo processo universale di trasformazione, al fine di inquadrare il lavoro terapeutico. Comunque, vi è una mancanza impressionante di una solida ricerca empirica su questi concetti junghiani, e c'è bisogno di uno sforzo comune, in psicologia analitica, per sviluppare le appropriate strategie di ricerca. Come esempio di questo genere di ricerca, vengono presentati i risultati del progetto della Structural Dream Analysis.

## Abstract (ru)

В статье обсуждаются научные предпосылки проведения международной конференции «Процесс трансформации», состоявшейся во Фрайбурге в октябре 2024 года. На основе тщательного анализа теории архетипов впервые было установлено, что в аналитической психологии существует путаница в вопросе определения архетипа. В действительности теория архетипов Юнга содержит не одну, а четыре теоретических основания. Так, в частности, биологические и антропологические аргументы Юнга можно считать игнорируемыми, в то время как идея универсальной формы процесса трансформации в психотерапии рассматривается более подробно. Юнг стремился составить карту универсального процесса трансформации, чтобы использовать ее в психотерапевтической практике. При этом наблюдается выраженный дефицит серьезных исследований этих концепций Юнга, и от аналитической психологии требуются согласованные усилия по разработки соответствующих исследовательских стратегий. В качестве примера такого исследования приводятся результаты исследовательского проекта «Структурный анализ сновидений».

## Abstract (es)

Este artículo presenta el fundamento científico para la introducción de la conferencia internacional sobre el «Proceso de Transformación» llevada a cabo en Friburgo en Octubre de 2024. Sobre la base de un análisis exhaustivo de la teoría de los arquetipos, se establece en primer lugar que existe confusión en cuanto a la definición de arquetipo en la psicología analítica. De hecho, la teoría de los arquetipos de Jung no contiene una, sino cuatro vertientes teóricas distintas. Mientras que los argumentos – en particular ‐ biologicistas y antropológicos de Jung deben considerarse refutados, la idea de una forma universal de un proceso de transformación en psicoterapia se examina con más detalle. Jung se esforzó por crear un mapa de este proceso de transformación universal con el fin de informar el trabajo psicoterapéutico. Sin embargo, hay una falta flagrante de investigación sólida sobre estas concepciones de Jung, y se requiere un esfuerzo concertado en la psicología analítica para desarrollar estrategias de investigación apropiadas. Como ejemplo de este tipo de investigación, se presentan los resultados del proyecto de investigación: Análisis Estructural de los Sueños.

## Abstract (zh)

转化的过程——分析心理学的核心以及如何对它进行研究

本文的内容为2024年10月在弗莱堡举行的“转化过程”国际会议提供了科学背景。经过对原型理论的深入分析, 文章首先提出分析心理学中关于原型定义的混乱。事实上, 荣格的原型理论并非只有一种, 而是包含四个不同的理论分支。值得一提的是, 尽管荣格的生物主义和人类学的论点必然已经不再具备说服力, 但对于心理治疗中普遍形式的转化过程的研究却愈加详尽。荣格努力为这一普遍的转化过程创建一张地图, 以指导心理治疗工作。然而, 荣格的这些观念缺乏坚实的研究, 分析心理学需要共同努力, 开发适当的研究策略。文章报告了结构性梦境分析研究项目的结果, 以此作为这种研究的例子。

## Introduction

The starting point for this conference was the investigation into archetype theory titled “Deconstructing Archetype Theory” (Roesler, 2023). This investigation produced a number of findings which have far‐reaching consequences for Analytical Psychology (AP), since archetype theory represents its core. First, there is confusion about the definition of archetype, and to this day no standard definition is available. Those that have been put forward are often incompatible or outright contradictory. At the heart of the problem lies the fact that Jung put forward a theory—and tried to defend it against any form of criticism, even if justified. His theory proposed that archetypes are embedded in the biological makeup of humans, like instincts/patterns of behaviour in animals, they have evolved in the prehistory of mankind, and because they are biologically rooted they appear “autochthonously” in every human being without any influence from culture and socialization; these biologically rooted archetypes are therefore responsible for similarities in the fields of religion, cultural patterns and social practices; finally, they have significant influence on the psychological development of the individual. In this theory, Jung attempted to bind together theories from biology, anthropology, religion, prehistory and mythology into one unified explanatory concept for the development of mankind, its cultures and religions as well as for the individual psyche—which is impossible. Jung’s theory must be characterized as a grandiose fantasy. It seems to me that this unifying approach is so fascinating and appealing that it is still highly attractive to many people, inside as well as outside of the Jungian community. As a result there is a strong tendency to cling to this belief system.

On the basis of a thorough analysis of Jung’s theorizing (Roesler, 2023), it becomes clear that there are different lines of thought which run through his theory of archetypes. As an attempt to present a solution to the confusion around archetype theory, I would like to propose the idea that in Jung—and in the debate around archetype theory in AP—we do not find just one coherent theory, but several distinct theories. These must be differentiated so as to create clarity:

## Theory 1: A Theory of Biologically Pre‐Formatted (Genetically Transmitted) Mental Capacities

According to this theory, humans are not a blank slate at birth. Due to their biological makeup, their genome and the similarity of their brain structure, certain pre‐formed features are innate. These can take the shape of instinctual behaviours/patterns of behaviour; preformatted categories that direct and form perception, and patterns which govern the formation of images, ideas etc. These patterns are thought to be transmitted from one generation to the other by way of the genetic code. They are considered to have the character of archaic behaviour and can appear primarily in regressive states of consciousness, especially in states of mental disturbance.

This is a biological theory, embedded in the natural sciences, and has strong connections to the medical disciplines, human genetics, neurosciences and ethology. Concepts from evolutionary theory are included, whereby humans are viewed as the product of a long line of evolutionary development, this being the reason why humans demonstrate archaic behaviour that can also be linked to our animal ancestors. Another associated idea is that there exists a “natural’ way of living for humans, a way of life in accordance with the background of our evolutionary history and development. In other words, a form of living which is “close to our nature”, fulfilling the needs implanted in our biological make‐up. This theory contains the idea that we can learn about this way of human life by looking back into the history of mankind.

## Theory 2: An Anthropological Theory of Human Universals

This theory belongs to the field of anthropology, and deals with the assumption of human universals that can be found in peoples from all over the world and from different epochs. The focus lies on similarities that are said to be found cross‐culturally in social rules and patterns, cultural habits and symbols/images, religious ideas, mythological motifs etc. This theory entails an idea characterized as the homology of phylogeny and ontogeny, meaning that the psychological development of the individual recapitulates the evolutionary and cultural development of mankind. Included in this idea is the assumption of a scale of developmental maturity from archaic/primitive to developed/civilized, which can accordingly be applied to the individual as well as cultural and societal development.

## Critique of Theories 1 and 2

In my book mentioned above, the latest research findings in anthropology, religious studies, archaeology and paleoanthropology were investigated and clearly demonstrate that the assumed similarities or primary forms, such as of religion, do not exist (for details see Roesler, 2023). Where there are parallels, e.g., in what anthropology has characterized as universals as well as in the field of comparative mythology, these can well be explained by migration, physical contact, cultural exchange, the interplay between regional environmental conditions and the dynamics inherent in culture and society itself (Witzel, 2012). Jung was dismissive of such viewpoints, because he was convinced that archetype theory was a biological theory and as such part of the natural sciences, which in his eyes was the only possibility to make this theory a truly scientific theory and defend it against critique. Ironically, this very approach has made archetype theory questionable, even unscientific, and has put it under massive critique (Papadopoulos, 1992). It has to be noted that Jung’s far‐reaching nomothetic statements about the universality of the so‐called great mother, and the hero myth connected with it, being images for the development of consciousness out of the unconscious, are not just some ideas in archetype theory, they are in fact at the centre of the architecture of the entire theory. The debate is not only about the existence or nonexistence of certain archetypes, but about the validity of Jung’s ideas forming a coherent explanatory system linking all the aspects in the unifying theory as presented above. All of these aspects play a role in the construction of the whole theory, they build a coherent architecture. In the face of the evidence speaking clearly against a universal distribution of these ideas and images, namely that of the so‐called great mother and the hero, the architecture of the whole of Jung’s archetype theory has practically collapsed. Although archetype theory, not only in the shape in which Jung presented it but also in present‐day form (see for example the teaching at institutes, introductory texts presented on the website of the International Association for Analytical Psychology, etc.), makes far‐reaching, even nomothetic statements about matters in the field of anthropology, religion and comparative mythology. Current archetype theory put forth in AP has lost contact with the development and state of scholarly knowledge in these fields or has even totally neglected it.

There is no doubt that human beings possess innate capacities, but these are the opposite of what Jung imagined to be biologically rooted archetypes: all of these innate elements are not structures and contents rather they are capabilities directed towards creating relationships, participating in communication, cooperation and sociality. In sum: they are directed towards social relationships. This is in contrast to what Jung imagined when he claimed a biological foundation of archetypes. The archetypes Jung had in mind cannot be conceptualized as biologically rooted in the face of contemporary knowledge. But still today, evidence for genetic pathways for mental capacities in humans are used to argue that such findings provide proof for Jung’s archetypes being biologically rooted (e.g., Goodwyn, 2020; Alcaro et al., 2017). Such authors still make the mistake to use such findings as “evidence” for the biological theory of archetypes, for conceptions of innatism or biological preformationism without taking into account that the archetypes of classical archetype theory—anima and animus, the wise old man, the trickster, the divine child, the journey of the hero etc.—are something substantially different from the capabilities that were found to be biologically rooted. Therefore, I would suggest not to use the term archetype anymore for biologically preformed mental propensities in humans, as this creates confusion instead of clarification. In sum, the first two aspects of archetype theory as outlined above have to be rejected as they are largely refuted by contemporary research. In contrast to this, a third line seems to be fruitful.

## Theory 3: A Transcendental Theory of a Unity Reality

This theory attempts to transcend the usual limits of normal science and bridge the gap between mind and matter by making use of concepts from quantum physics. These ideas were mainly developed in the Pauli‐Jung‐dialogue (Roesler & Reefschläger, 2022). A product of this conversation is the concept of synchronicity and the idea of *unus mundus*: a potential reality in which mind and matter are still united. These ideas had a strong influence on the development of transpersonal psychologies, contemporary consciousness studies, and spiritually integrated therapies (Roesler & Reefschläger, 2022). In this regard these speculations resulted in being very fruitful for different academic fields.

## Theory 4: A Process Theory of Psychological Transformation

They main topic of the present paper, though, is the last part inherent in Jung’s theory. It is concerned with describing transformational processes, which can be observed in psychotherapy as well as in other forms of transformational phases in human life. The general idea of this theory is the assumption of a force within the human psyche, which is behind this process of psychological transformation and aims at greater integration of the personality, whereby this integration of the totality of a person, called wholeness, is preformatted in the unconscious—the Self. There is a universal form of the transformational process towards integration, applicable to all human beings, and can, if unfolded, be used as a map for the psychotherapeutic process. Models for this universal map of transformational processes in the psyche can be found in symbolic form in certain cultural and religious traditions, mystical teachings (e.g., Gnosis, Yoga, the Buddhist path etc.), religious scriptures, in alchemy as well as in mythologies and fairy tales. One of Jung’s major aims was to create a map of this universal process of transformation, so as to be applied in the practice of psychotherapy. The archetypes, which Jung provides a detailed description of, and which I have called the “classic archetypes” (anima/animus, the shadow, the wise old man, the great mother, the self, the trickster, etc.; see Roesler, 2021), were conceptualized as being stages of this process. It is assumed that this process and its stages are preformatted in the psyche of every individual, throughout the world, during every epoch. Training in Jungian institutes is concerned mainly with creating a deep understanding of this transformational process, and research and much activity around publications in the Jungian world contribute to establishing a map of this process. This theory is the core of AP. This idea is a unique contribution of Jung’s to the development of a theory of psychotherapy. He was the first to present this idea, which had a strong impact on the formation of other psychotherapy schools, particularly of humanistic and transpersonal approaches. It should be noted, however, that there are different versions of the transformational process.

## The Process Idea

*For years I have been observing and investigating the products of the unconscious in the widest sense of the word, namely dreams, fantasies, visions, and delusions of the insane…. There are types of situations and types of figures that repeat themselves frequently and have a corresponding meaning …. [These] can be arranged under a series of archetypes, the chief of them being … the shadow, the wise old man, the child (including the child hero), the mother (“Primordial Mother” and “Earth Mother”) as a supraordinate personality, and her counterpart the maiden, and lastly the anima in man and the animus in woman*. (Jung, 1959a, para. 309)

For Jung, the idea of the individuation process provided the theoretical model for the practice of psychotherapy. This process appears as if there were an autonomous factor in the psyche which brings about the transformation (Jung, 1959a, para. 282). The archetypes represent a totality or an image of wholeness of the personality (Jung, 1959a, para. 293). This process results in a synthesis of consciousness and the unconscious (Jung, 1959a, para. 297; Jung, 1969, paras. 400–411).

Nevertheless, there seem to be at least four different models of this process inherent in Jung’s theorizing to be differentiated. *First*, Jung characterizes this as a centring process, for which the archetype of the Self is responsible, and which can manifest itself in images in the shape of mandalas in the broadest sense (Jung, 1953, para. 260). *Second,* the idea of this process contains a model of a sequence of stages which are clearly defined (the shadow, anima and animus, the wise old man/the great mother, etc.; Roesler, 2021). The latter is a theory of a much higher complexity, and makes far‐reaching claims in the sense of nomothetic statements, whereas the first theory would probably find agreement among a number of contemporary schools of psychotherapy, e.g., Rogers’ approach or systemic schools, as it contains the idea of a self‐organizing nature of the psyche. *The third model*, again a process of stages, contains as the central element the idea that consciousness develops out of an unconscious matrix which is pictured in myths of the hero emancipating from the great mother (Jung, 1969, para. 415; Neumann, 1966; the hero’s journey later elaborated by Campbell, 1971). There are a number of problems with this model: first, the development of the brain cannot be described as starting from an unconscious matrix. On the contrary, consciousness is first. Second, Jung and Neumann chose their material very selectively and took a Eurocentric view, the idea of a great mother as the source of life being by no means universal (Roesler, 2023).

Finally, there is a *fourth model,* using the imagery of alchemy, as Jung found that the alchemists had attempted to describe such a process of transformation of the psyche in metaphors of chemical substances and their transformations:

Nigredo, for the dark night of the soul, when an individual confronts the shadow with it; separatio, for the moment of emotional and spiritual discrimination; mortification or putrefactio, for the stage at which the old neurotic ways of being are cast off; dissolutio, for the initial disorientation after the old self is discarded. (Hopcke, 1989, p. 165)

In general, it can be said that in Jung’s idea of the individuation process, the archetypes form a sequence, most clearly described in “The Relations Between the Ego and the Unconscious” (Jung, 1953): whereas initially the ego identifies with the persona, in the transformation process it must deal with its counterpart, the shadow. If this is accomplished, the ego meets anima/animus and must establish a relationship to the soul. On the further road, the ego will meet the wise old man and the great mother (the Mana‐personalities), which surround the archetype of the Self. The divine child often appears at moments where transformational processes take place, because it symbolizes the new hope for the future. The trickster is a helpful figure, sometimes accomplishing tasks through tricks and twists, which the ego cannot overcome. The *coniunctio* is closely related to the realization of the Self, which is symbolized in mandala‐like figures or symbolisms of wholeness, completion etc.

## The Process of Transformation in Psychotherapy

Jung’s major interest in establishing his psychology was to create a map of the process of transformation as he saw it, therefore his interest in alchemy, the study of religions and mythology. The idea is that the archetypes, which shape the process, are also expressed in the form of mythological motifs and narratives of myths and fairy tales of peoples of the world (Jung, 1959a, para. 260). If the map of this process and its stages were available, elements of the process could be detected in material provided by patients in analyses, e.g., in dreams (e.g., Jung, 1959b, para. 208). A widespread use of this model in AP is to make an association between an image or symbol within the dream of a client and a fairy tale or other mythological story (Roesler, 2021). The general idea, put more technically, is that the unconscious makes a connection to an archetypal pattern, which is spelt out in the mythological story in symbolical form, and which contains information, in addition to the conscious material that client and therapist already hold that is helpful for the therapeutic process. The Jungian therapist relies on the belief that the whole of archetypal information is potentially accessible to any of their clients via the (collective) unconscious and can be activated under suitable circumstances. This means that a concept of *universal* archetypes is necessary for AP, as otherwise, if we could not count on this, we would be unable to work as we do. So, this process idea forms the core of AP and its clinical practice.

It is interesting that recent accounts of the process, which is assumed to unfold in Jungian psychotherapy, tend to ignore the model of the stages containing the classic archetypes. For example, Kast (1992) successfully integrated the insights of infant observation (Stern, 1985), but describes the psychotherapeutic process, although she calls it archetypal, without any reference to the classic archetypes. The same applies to Mario Jacoby (1998), who integrated the Jungian approach with Kohutian self‐psychology. The only reference to the term “archetype” is to the “creative and ordering factor, which is called archetype” (p. 89). The conceptualization of the “self’ follows Stern (1985), not Jung.

## Is Jung’s Model of the Individuation Process Universal?

There are two major problems connected with these ideas of a universal transformational process: the first being the question of validity of these concepts, and how Jung came to conceptualize this process in the form he presented it; the second question concerns the role of the therapeutic relationship in this process.

Jung claims that he developed his model of the process in psychotherapy from clinical material provided by his patients (Jung, 1969, para. 401). This is what I have termed the model of a centring process, in contrast to the model which includes all the stages represented by the classic archetypes. As to the second model, it seems to me that he developed these ideas during his so‐called confrontation with the unconscious as part of the crisis after the break with Freud. So, the question arises: did Jung elaborate this model just from his personal experiences, and from then on applied it to his cases in the conviction that what he had experienced was universal?

Having once declared a theoretical system of explanation as valid for oneself, it will form the way how we look at reality. So, there is the danger of constructing a reality which is based more on one’s own concepts than on the patient’s reality. In the history of AP, starting with Jung himself, there has been notable effort to find confirmation for Jung’s theory. What is missing, from my point of view, is a more sceptical attitude, and with it an active search for confirmation or refutation of the concepts. Because of this attitude, Jung’s case examples as well as many others published in the history of AP may have served the aim, first and foremost, to affirm the already existing model, instead of investigating it open‐mindedly. Westen & Morrison (2001) point out the problems associated with the dominant research tradition in psychoanalysis which is based mainly on case reports:

Narrative case reports … are invariably compromise formations. We hope they include a heavy dose of relatively accurate perception and memory. But as compromise formations, they are likely to reflect a variety of wishes and fears. Convincingly, to appear intelligent and clinically talented to one’s colleagues, to establish one’s identity as a member of the analytic community (or a subset of it), to express identification with admired others and with those whose admiration one desires, to express competitive or hostile impulses toward those with whom one disagrees or dislikes, and so forth. Among the most important limitations are lack of replicability, lack of reliability of inference, lack of control over variables that would allow causal inference, and unknown generalizability. (Westen & Morrison, 2001, p. 883)

When a critical attitude was applied, it could be demonstrated that even classical cases of Jung’s could be well explained without any reference to archetypes (e.g., Merchant, 2019). But it seems to me as if predominantly ideal therapies are being passed on and remembered in AP. When looking at first‐hand reports by former clients of Jung’s (for a detailed account see Roesler, 2023), it is stunning how Jung did not even occupy himself with the material of the patient but rather held lectures, presenting his theory and how it had to be applied to the patient (e.g., Reid, 2001). What these reports tell us is that Jung, absolutely convinced of the validity of his concepts, was willing to force them onto his patients, instead of dealing with the material in an unprejudiced manner (Bair, 2003; Healy, 2017). What I am trying to demonstrate is that we should be careful regarding the clinical material Jung provides as foundation for his theory (see also Roesler, in review). As a result of these considerations, this calls for a new initiative to investigate the process of transformation in psychotherapy with a more open‐minded attitude and adequate methodologies.

## Transformational Processes as Topic of Psychotherapy Research

The question of how transformation in psychotherapy comes about and how the transformational processes can be described in detail, is a major topic in contemporary psychotherapy research (e.g., Castonguay & Hill, 2012). This research attempts to identify principles of change at work in psychotherapeutic processes. What has to be pointed out is that the major factors of change identified here are connected with the therapeutic alliance in which so‐called corrective emotional experiences take place. In psychodynamic (and also humanistic) approaches this always means a new and healing experience in the therapeutic (transference) relationship. This contrasts with Jung’s viewpoint: in contemporary approaches the emphasis is on the relationship whereas with Jung the process comes from within the person. In Jung’s perspective, development of the personality happens almost exclusively from inside the individual, autonomously; relationships do not play a role beyond their being a projection screen. 2

This attitude is in drastic contrast to the current research in human and social sciences—and in practically all the other psychodynamic schools—in that relationships are at the very beginning of individual development and are absolutely essential for the development of the personality (see Chapter 5 in Roesler, 2023). Knox (2009) points out that all of the contemporary relational approaches in psychoanalysis agree on the three fundamental developmental tasks that have to be accomplished in the process of therapy:

1. affect regulation
2. the capacity for mentalization
3. a sense of self agency

These capacities directly result from the experience of the relationship with the therapist (for a detailed discussion see Roesler, 2024a). These tasks are very different from what Jung thought to be the aims of therapy. Here, the relational model speaks of mental capacities, whereas Jung talks about clearly defined stages of a process. It also has to be noted that, based on research which found that inborn capacities consist of interaction and relationship abilities, in contemporary psychodynamic approaches the self in development is always conceptualized as “Self‐being‐with‐other” (Stern, 1985)—which is fundamentally different from Jungian conceptualizations. Contemporary approaches see the relationship at the beginning, whereas the Jungian school assumes that the Self is preformatted and primary. This is an unresolved question in AP: where does development and therapeutic change come from, is it the experience of the relationship with the therapist (which is then internalized as “good object”), or are the therapist and the therapeutic relationship just working as catalysts for an autonomous process coming from within?

Speaking of how change is effected in psychotherapy: there is another process model, or better to say, a process metaphor, inherent in Jungian theorizing, which is rarely mentioned but, from my point of view, probably the most important metaphor for psychotherapeutic change: Jung’s contribution of the idea of “death and renewal” being a basic image of how psychological change comes about, namely by letting go of the aims of the conscious ego—Jung sometimes uses the term sacrifice—which makes change and renewal possible. Personally, I believe that this is the most important gift Jung made to psychology, as it refers to the deep mystery of how change in human life is accomplished, and it also links Jungian psychology with the religious and especially the mystical traditions. It also distinguishes Jungian psychotherapy from all the other psychotherapeutic schools and puts it in a spiritual context (Roesler & Reefschläger, 2022).

## What Kind of Transformational Process?

I believe that the idea of an archetypal process inherent in Jung’s archetype theory can still be used for the psychotherapeutic process, but this requires that we give up on the biologistic and nomothetic, in many aspects even positivistic statements that Jung made about archetypes. In contrast, the theory of an archetypal process which takes place in psychological transformations, and which can be described by its stages, has to be regarded as an interpretation schema, a template for what could be called a clinically applied hermeneutics.

Consequently, a properly Jungian hermeneutics involves the deployment of a flexible (pluralistic), comparative, and interdisciplinary exegesis that seeks out interpretive possibilities—not conclusions—and whose canonic procedures amplify the symbol‐text by adding to it a wealth of personal and collective, historical and cultural analogies, correspondences, and parallels. In other words, the Jungian interpretation unfolds as a production—a positing of meanings in relation to and not the uncovering of “the meaning”. (Barnaby & D’Acierno, 1990, p. XVII)

This contrasts with what could be called “vulgar Jungianism (the mechanical and reductivist allegorical rewriting of a text according to the master code of the archetypes)” (Barnaby & D’Acierno, 1990, p. XXI). Rather, we have to discard the idea that the meaning is fixed to the symbol, an idea which can only be characterized as a primitive form of naïve essentialist epistemology. Instead, meaning is only produced in an interactive relationship between at least two human minds—this being the reason why psychotherapeutic change needs two persons. And this relationship is much more than just a projection screen, it is a place where something new emerges. I believe that here, still, all the wonderful imagery of alchemy can be applied, as has been brilliantly pointed out by Nathan Schwartz‐Salant (1998). But we always have to keep in mind that these images are rather than tools to uncover “the meaning”. Jung’s way of describing the transformational process in terms of archetypal forms and images could be reformulated as being a theory of cultural symbolization processes of psychological transformations.

There is no alternative to discarding the majority of assumptions inherent in archetype theory about the “instinctual” foundation of the psyche or any other dubious biological conceptualizations as well as assumptions about transcultural parallels. The theory of an archetypal process of change can only survive if we radically reduce the theory to an explanatory model for the process of psychotherapy. There is a deep truth in the idea that there is a universal and autonomous process in the psyche unfolding over the course of psychotherapy. I also believe that this process can be mapped. I am quite sceptical, though, whether this map, when we have investigated well‐documented processes open‐mindedly, will look like what Jung described.

But the general idea Jung proposed, that there is a helping force in the unconscious which supports the therapeutic process by presenting symbols, images and narrative patterns, e.g., in dreams, I see as one of the most important contributions to the field of psychotherapy in the 20^th^ century. I would also stress the point that we can work with this approach without making dubious statements about its biological foundation, or by drawing questionable parallels to the fields of anthropology, religion, prehistory and the like.

## Desperately Seeking Research

These conclusions imply specific directions for future research in AP. There is especially a great need to conduct research on psychotherapeutic processes. The crucial point is to establish a comprehensive and standardized system of documentation to be applied to psychotherapeutic processes, so as to create a databank which would allow for detailed investigations of unconscious dynamics going on in psychotherapy. Only if we succeed in creating such detailed documentations, will we be able to search for inter‐individual patterns, structures, symbols, processes etc., which then could support the above‐mentioned idea of a universal process taking place in psychotherapy—which we may then call archetypal. As I have argued in many places, what is needed to create such research approaches is, on the one hand, a more open‐minded attitude in Jungians, which is not fixated on providing proof for Jung’s theories whatever it may cost, but instead is interested in finding out how things really are—in this case, what really happens during the course of psychotherapy. On the other hand, as soon as this attitude is given, we need a collective (joint) effort to collect data, to systematically document our psychotherapies, and to build a solid databank for such investigations into the process of psychotherapy.

Steps towards such research have already been taken in Germany and Switzerland. In the Base Documentation (BADO) study conducted in Germany candidates in training had to apply a set of scales to their training cases. The questionnaires’ measures cover the main areas of patient problems/resources: socio‐demographic variables, symptoms, personality structure and sense of meaning in life. The scales are applied at initiation of therapy (T1) and termination of therapy (T2), and, if possible, at follow‐up (T3). In this naturalistic outcome study, more than 100 cases have been documented, the results will soon be published. Additionally, therapists documented information about the patient, the psychodynamics and the course of therapy using a systematized single case reporting frame, so that inferences can be made between the results of therapy and personality, conflicts and complexes of the patient. Finally, therapists were asked to collect all the dreams documented over the course of therapy as well as symbolic material, if available. These additional data will allow for deeper interpretations regarding the connection between symbols and images appearing over the course of therapy and therapeutic change. A similar project has been started with data collection by experienced analysts (OPA‐P, see www.infap3.eu/forschungen).

As an example for such research, I will present some findings from the Structural Dream Analysis research project.

## Psychotherapy Process Research in the Context of Structural Dream Analysis

Structural Dream Analysis (hereafter SDA, Roesler, 2018a) assumes that the meaning of dreams is not so much transmitted by the elements or symbols in the dream but by their interrelationships, or “structure’, especially the relationship of the dream ego to other elements in the dream, and the extent of agency of the dream ego. This approach follows Jung’s idea of interpretation on the subjective level. SDA views the dream as a narrative, a short story about how the protagonist, in most cases the dream ego, addresses a problem. Based on a number of extensive single case analyses, a typology of dream patterns was created as well as a theoretical model of the correlations between dreams/dream patterns, initial psychopathology, and the course of psychotherapy (Roesler, 2018b). The dream patterns represent the general idea of SDA in that the dream forms a micro‐narrative in which a problem is presented that the dream ego has to struggle with. The ego is confronted with a challenge and attempts to fulfil a plan or task. The agency of the dream ego rises continually, starting from Pattern 1 (no ego present at all) to Pattern 2 (dream ego is threatened) and then to Pattern 3, in which the dream ego has to perform a task but is under pressure from other forces whereby the initiative is not with the ego but under their power and control. In Pattern 4 (dreams of mobility) and 5, the ego has taken over the initiative and follows a personal plan. On the level of Pattern 5, the focus is on creating satisfying relationships with others. Finally, at Pattern 6, the dream ego gains full autonomy from other elements and can operate freely.

These dream patterns were found not only inter‐individually, but also cross‐culturally (Roesler, 2024b). This result is interesting insofar as no specific symbols or motifs were found to be universal except for specific patterns in which the dream ego responds to a problem or challenge, and the degree of agency that the ego takes on.

Dream patterns can be interpreted psychologically as an expression of the capacity of the dreamer’s ego, on different levels, to regulate and cope with unresolved complexes, something that psychoanalysis calls ego strength (Roesler, 2024b). In those cases where psychotherapy was successful, as evidenced by an improvement in symptoms, psychological well‐being, emotional regulation and, from a psychoanalytic point of view, a gain in psychological integration and ego strength, we found a typical pattern of transformation in the structure of their dreams. Characteristically, the initial phase of the psychotherapeutic process was dominated by a repetitive pattern in the patient’s dreams, which showed a weak dream ego incapable of solving the problem presented in the dream, such as being threatened without a strategy to cope with the danger, other than fleeing or attempting to hide. In Pattern 3 dreams, where the dream ego has to fulfil a task, it typically fails, is not prepared, acts too late, etc. In mobility dreams (Pattern 4), the dream ego typically fails to reach the desired aim, is on the wrong bus or train, or has no ticket, etc. If psychotherapy is successful, the typical patterns change into more successful activities of the dream ego: it confronts threatening figures, fights actively, and successfully overcomes the threat, fulfils the task (e.g., passes an exam) or succeeds in reaching the desired aim.

In general, there is a movement from lower patterns (1, 2 and 3) dominating the first half of the dream series, where the dream ego is subjected to others’ initiative or feels threatened, towards patterns 4, 5 and 6 in the second half of the dream series, where the dream ego gains more and more agency and solves the problem successfully. For instance, it may be more capable of creating satisfying interactions with others, including sexual encounters, or makes itself more independent from others. This transformation is interpreted from a psychodynamic perspective as speaking to the fact that an initially weak ego structure, which fails to regulate and integrate threatening complexes, gains in ego strength over the course of the therapy and succeeds in coping with initially repressed or split off parts of the psyche, integrating them into constructive interactions with others. So, the relationship between the dream ego and threatening figures, and the reaction of the dream ego to the threat, reflects the relationship between actual ego strength and unintegrated or conflicted parts of the psyche (complexes). The particular image in which the threatening figure appears—especially if the dream pattern is repetitive—can be seen as symbolizing the psychological problem, the complex with which the dreamer is struggling. On the other hand, we found no support for the assumption that there are typical or even fixed meanings for specific symbols. For example, in one case of a female dreamer, the dream ego was repeatedly threatened by snakes. The therapist diagnosed an unresolved conflict between a highly moralistic superego on the one hand and very lively sexual desires on the other. The snake here can be interpreted as a sexual, phallic symbol, which appears threatening to an ego under the pressure of the moralistic superego. In contrast, in the dreams of a young man, the snake repeatedly acted as a helper.

On the other hand, in a number of dream series we found a typical motif appearing at turning points in psychotherapy whereby after a repetitive pattern of dreams with the ego being threatened, it finally overcame the danger. This reflected a sudden leap from lower to higher dream patterns. At these pivotal moments the motif of a child or baby appeared, which the dream ego has to care for, and which is often born under mysterious circumstances (e.g., also men can become pregnant and give birth to the child). Of course, a Jungian here is reminded of Jung’s discussion of the archetype of the divine child (Jung, 1959a), and in fact dream children we found have many of the characteristics Jung attributes to the divine child. The following example of such a dream marked this change in a six‐year‐analysis of a young man:

A little baby is in danger. I cover it with newspaper and carry it with me through a sewage system. Then I forget about it and leave it somewhere. But then I realize that the baby is missing and go back and find it again. I carry it with me and feed it. I think, “the baby is so small, it should get mother’s milk”, but I can just feed him solid food.

Thus, this is empirical evidence of a true archetypal motif, which marks turning points in the process of transformation, and which is completely unconscious to the dreamer (publication forthcoming). These findings from dream research could serve as an example to demonstrate that it is possible to investigate Jungian models of the process of transformation if carefully documented clinical material and data are available and suitable research methods are applied.

## Footnotes

^2^
For example: “At the heart of marriage is the question whether one can live his true nature, and if one can give the other—being it husband or wife—the freedom for their individuation” (Jung in Jaffé, 1989, p. 57; ). This means a relationship, such as marriage, at best, is not an obstacle to individuation.
